# Cognitive impairment and hospital admission risk among community-dwelling adults in the MAUCO cohort

**DOI:** 10.1080/07853890.2026.2716525

**Published:** 2026-08-20

**Authors:** Javiera Leniz, Nicolás Valdés, Valentina Rios, Laura Huidobro, Sandra Cortés, Claudia Bambs, Catterina Ferreccio R., Pablo Toro

**Affiliations:** ^a^ Escuela de Salud Pública, Facultad de Medicina, Pontificia Universidad Católica de Chile, Santiago, Chile; ^b^ Advanced Center for Chronic Diseases, ACCDiS, Santiago, Chile; ^c^ Escuela de Medicina, Facultad de Medicina, Pontificia Universidad Católica de Chile, Santiago, Chile; ^d^ Facultad de Medicina, Universidad Católica del Maule, Talca, Chile; ^e^ Center for Cancer Prevention and Control, CECAN FONDAP 152220002, Santiago, Chile; ^f^ Instituto de Salud Pública de Chile, Ministerio de Salud, Santiago, Chile; ^g^ Departamento de Psiquiatría, Escuela de Medicina, Facultad de Medicina, Pontificia Universidad Católica de Chile, Santiago, Chile

**Keywords:** Mild cognitive impairment, hospitalization, hospital admission, readmissions

## Abstract

**Background:**

Understanding whether cognitive impairment increases the risk of hospital admissions is essential for preventing burdensome care. However, most evidence focuses on people with dementia diagnosis and comes primarily from Europe and North America.

**Objective:**

To evaluate the association between screening-based cognitive impairment and the incidence of hospital admissions and re-admissions, and to identify factors associated with hospital admission risk in the MAUCO cohort, the first prospective cohort from central-southern Chile.

**Materials and methods:**

A prospective population-based study including adults (38–74 years) from the MAUCO cohort with a Mini-Mental State Examination (MMSE) and/or an Addenbrooke’s Cognitive Examination-Revised (ACE-R) test at baseline. Hospital admissions were identified through administrative records. Multivariate Cox and negative binomial regressions with Inverse Probability Treatment Weighting were used to estimate associations between cognitive impairment and (1) hospital admission (2), 30-day all-cause readmission and (3) number of hospital admissions.

**Results:**

Of 9066 participants with an MMSE, 6776 also completed an ACE-R. Mean age was 53.3 years, 54.5% female, and 19.8% had less than six years of education. Cognitive impairment prevalence was 19.7% by MMSE and 19.3% by ACE-R. Over a mean 7.8 years follow-up, 23.1% had 1+ hospital admissions, and 12.1% of them experienced a readmission. People with MMSE-defined cognitive impairment had a 40% higher incidence rate of hospital admissions than age- and sex-weighted controls without impairment (IRR 1.40; 95% CI 1.18-1.66), but readmission rates did not differ. Diabetes and self‑reported health status were the strongestpredictors of hospital admissions and readmissions, whereas associations with cognitive status varied by cognitive instrument, impairment category, and hospital admission outcome.

**Conclusions:**

MMSE-based cognitive impairment was associated with higher hospital admission rates, whereasACE-R-based imparimen showed a less clear pattern. Chronic conditions and disease burden may play a more important drivers of hospital use than cognitive status alone.

## Introduction

Cognitive disorders represent a growing public health challenge worldwide due to population ageing and increasing life expectancy. Cognitive impairment encompasses a spectrum of conditions ranging from mild deficits in cognitive performance to more severe cognitive and functional impairment suggestive of major neurocognitive disorder or dementia [1]. Globally, cognitive impairment affects approximately 12–15% of community-dwelling adults aged 50 years and older, with prevalence increasing to about 20% among those aged 60 years and older [2]. Dementia, the most severe manifestation of cognitive impairment, affected approximately 57 million people worldwide in 2019, with over 60% residing in low- and middle-income countries, and nearly 10 million new cases reported every year [3]. While the association between age and cognitive function is well established, increasing evidence suggests that subtle cognitive decline may be observed during midlife, several years before the clinical diagnosis of cognitive impairment or dementia [4,5].

Cognitive impairment has been associated with increased vulnerability to adverse health outcomes and healthcare use, such as a higher risk of mortality and hospital admissions [6]. Hospital admissions may be particularly challenging for people with cognitive impairment and dementia, which may worsen their cognitive status and functional decline, increase their risk of adverse drug reactions [7], delirium [7], pressure ulcers and falls [8–11]. Cognitive impairment has also been associated with a higher incidence of institutionalization [12], and hospital readmission after discharge [13].

Most of this data comes from high-income regions, such as the United States, Canada and European countries, revealing a substantial gap in evidence from low- and middle-income and Latin American countries, with only one study in Brazil that found an increased probability of hospital admission, with a 95% greater risk compared with individuals with preserved cognition [14]. In addition, research in this area has focused largely on older adults, whereas the consequences of cognitive impairment in middle-aged populations remain poorly understood. Given that cognitive decline may begin years before the onset of clinically recognized dementia, understanding its impact across the adult life course is increasingly important.

Chile is experiencing a rapid demographic transition, with a growing proportion of older adults and an increasing burden of cognitive disorders. Recent estimates suggest that approximately 174,921 people were living with dementia in 2019, a number projected to double by 2050 [3]. Despite these trends, evidence on the health consequences of cognitive impairment in Chile remains scarce, particularly among community-dwelling populations and middle-aged adults. Understanding the relationship between cognitive impairment and hospital admissions in the Chilean context is essential for informing prevention strategies, planning healthcare services, and anticipating future demands on the health system associated with population ageing.

We aimed to evaluate the incidence of hospital admissions and re-admissions among individuals with cognitive impairment based on screening tests compared with those without cognitive impairment, and to identify factors associated with hospital admission risk in the MAUCO cohort, a large population-based prospective study from Chile, thereby contributing local evidence from an underrepresented Latin American population and informing the development of prevention strategies for adverse outcomes among community-dwelling adults with cognitive impairment.

## Materials and methods

### Study design and data sources

This study is part of the MAUCO (Maule Cohort) project, a population-based prospective cohort study conducted in Molina, a semi-rural city located in the south-central region of Chile. Participants were recruited between 2014 and 2019 using household census-based sampling. Adults aged 38 to 74 years old residing in Molina were eligible for inclusion. We recruited 9462 participants who provided self-reported sociodemographic, health status, lifestyle, psychosocial and cognitive symptoms that were collected at baseline, in addition to undergoing biometric measurements and laboratory tests.

### Ethical statement

The MAUCO study protocol was approved by Ethics Committees at Pontificia Universidad Católica de Chile (N◦ 14–141) and the Maule Regional Service of the Chilean Ministry of Health. Written informed consent was obtained from all MAUCO participants [15,16].

### Study population

The study population consisted of MAUCO participants aged 38–74 years who underwent a baseline cognitive assessment using a Mini-Mental State Examination (MMSE).

### Assessment of cognitive function

All participants were evaluated using the Addenbrooke’s Cognitive Examination-Revised (ACE-R), which includes the MMSE test as part of its evaluation. Both instruments are validated in the Chilean population [17,18]. Assessments were administered by nurse technicians trained by an expert psychiatrist.

The ACE-R is widely used because of its greater sensitivity and predictive value in detecting dementia in comparison to MMSE. The ACE-R incorporates the MMSE within its structure, allowing scores for both instruments to be derived simultaneously. The score ranges from 0 to 100. Participants were classified as cognitively unimpaired if they had an ACE-R score > = ACE-R score mean −1 SD, mild cognitive impairment (MCI) between ACE-R score mean −1 SD and −2SD, or suggestive of dementia with an ACE-R score < ACE-R score mean −2 SD.

The MMSE is a validated screening tool for cognitive impairment, with scores ranging from 0 to 30 [19]. Because MMSE performance is influenced by educational level, education-adjusted cut-off points were applied to define cognitive impairment: ≤22 for fewer than 5 years of education, ≤23 for 6–11 years, and ≤24 for those with at least 12 years of education, as proposed by Kochhann et al. [20]. Participants with a missing value on one subsection of the MMSE but a total score above the cut-off point were classified as cognitively unimpaired.

As some participants had incomplete ACE-R assessments due to missing responses on one or more items, MMSE and ACE-R were analysed separately, resulting in two analytical samples.

### Outcome

The study outcomes were:

1. Time prior to first hospital admission during follow-up
2. Number of hospital admissions during the follow-up
3. 30-day all-cause hospital readmissions

To identify hospital admissions, the MAUCO project established formal agreements with public hospitals in the region where participants are most likely to receive care. These agreements allow linking individuals admitted to hospital with MAUCO’s database, automatically generating reports for the surveillance team, which include the date of admission, length of stay and cause of hospital admission. We included admissions for all causes except maternal causes. Data was last updated in January 2024 prior to analysis.

### Covariates

Age (<60 and ≥60 years old) and years of education (<6; 6–8, >8 years) at baseline were categorized only for descriptive purposes but analyzed as continuous variables. Medicines reported by participants at the time of the baseline interview were converted to Anatomical Therapeutic Chemical (ATC) codes by a trained pharmacist. After excluding homoeopathic medicines and wrong codes, the number of unique ATC codes reported for each participant was considered. Self-reported health status was assessed using the question ‘Would you say your health is excellent, good, regular or bad?’, and was categorized as excellent-good or regular-bad.

Cardiovascular risk factors were assessed through self-report, biometrics and laboratory results. Participants with hypertension were identified through self-reported diagnosis or antihypertensive medication use, or an average blood pressure > =140/90 mmHg at baseline. Participants with diabetes mellitus were identified through self-reported diagnosis or hypoglycaemic drug use, a fasting blood glucose level > =126 mg/dl, random blood glucose > =200 mg/dl or a glycated haemoglobin > =6.5%. Smoking behaviour was categorized as current, former, or never smoked.

The Patient Health Questionnaire (PHQ-2), a validated brief version of the PHQ-9 for early detection of depressive disorders, was applied at baseline. We used the recommended cut-off point of > =3 in the PHQ-2 score to define participants at risk of depressive disorders [21].

#### Data/statistical analysis

The cumulative probability of having a hospital admission and a re-admission to hospital within 30 days of discharge for people with and without cognitive impairment based on the ACE and MMSE score (control vs cognitive impairment) was analyzed using a Kaplan-Meier estimation.

The association between the level of cognitive impairment based on ACE score and MMSE score and the risk of having a hospital admission and a 30-day re-admission were assessed using Cox proportional hazards regression models. We used Inverse Probability of Treatment Weighting (IPTW) estimated using logistic regression to balance age and sex differences between ACE-R and MMSE categories and weighted analyses were used to estimate the average treatment effect [22]. We evaluated the balance before and after IPTW across ACE-R and MMSE categories using a density plot and bar graphs for age and sex, respectively. Results from IPTW are reported in (Supplementary material Figures S2 and S3, and tables S1 and S2).

**Table 1.** Sociodemographic characteristics of the entire sample and by category of ACE-R and MMSE scores.

		ACE-R				MMSE		
	TOTAL	Unimpaired	MCD	Suggestive of dementia	p-value	Unimpaired	Cognitive impairment	
	(*N* = 9066)	(*N* = 5471)	(*N* = 943)	(*N* = 362)		(*N* = 7287)	(*N* = 1779)	*p*-Value
Age at baseline; Mean (SD)	53.3 (9.76)	52.5 (9.64)	54.8 (9.54)	56.8 (9.57)	<0.001	52.2 (9.57)	57.7 (9.27)	<0.001
Age at baseline categorical								
<60	6486 (71.5%)	4081 (74.6%)	638 (67.7%)	208 (57.5%)	<0.001	5508 (75.6%)	978 (55.0%)	<0.001
> =60	2580 (28.5%)	1390 (25.4%)	305 (32.3%)	154 (42.5%)		1779 (24.4%)	801 (45.0%)	
Sex (Female)	4937 (54.5%)	2993 (54.7%)	452 (47.9%)	187 (51.7%)	0.001	4001 (54.9%)	936 (52.6%)	0.22
Marital status								
Married or living partner	6035 (66.6%)	3724 (68.1%)	606 (64.3%)	231 (63.8%)	0.015	4948 (67.9%)	1087 (61.1%)	<0.001
Divorced or widowed	2644 (29.2%)	1506 (27.5%)	297 (31.5%)	122 (33.7%)		2001 (27.5%)	643 (36.1%)	
Missing	387 (4.3%)	241 (4.4%)	40 (4.2%)	9 (2.5%)		338 (4.6%)	49 (2.8%)	
Years of education; Mean (SD)	9.06 (4.03)	9.45 (3.96)	8.59 (3.74)	11.7 (52.1)	<0.001	9.95 (3.58)	5.45 (3.75)	<0.001
Missing	31 (0.3%)					31 (0.4%)	0 (0%)	
Years of education categorical								
<6 years	1797 (19.8%)	998 (18.2%)	173 (18.3%)	38 (10.5%)	0.009	852 (11.7%)	945 (53.1%)	<0.001
6 a 8 years	2524 (27.8%)	1520 (27.8%)	266 (28.2%)	96 (26.5%)		2004 (27.5%)	520 (29.2%)	
>8 years	4714 (52.0%)	2953 (54.0%)	504 (53.4%)	228 (63.0%)		4400 (60.4%)	314 (17.7%)	
Missing	31 (0.3%)					31 (0.4%)	0 (0%)	
Monthly income								
<US$194.9	2535 (28.0%)	1431 (26.2%)	266 (28.2%)	120 (33.1%)	0.077	1872 (25.7%)	663 (37.3%)	<0.001
US$194.9 to US$355.6	1971 (21.7%)	1150 (21.0%)	204 (21.6%)	70 (19.3%)		1527 (21.0%)	444 (25.0%)	
> =US$355.6	4485 (49.5%)	2851 (52.1%)	460 (48.8%)	167 (46.1%)		3827 (52.5%)	658 (37.0%)	
Missing	75 (0.8%)	39 (0.7%)	13 (1.4%)	5 (1.4%)		61 (0.8%)	14 (0.8%)	
Self-reported health status								
Excellent/Good	4072 (44.9%)	2650 (48.4%)	345 (36.6%)	125 (34.5%)	<0.001	3522 (48.3%)	550 (30.9%)	<0.001
Regular/Bad	4968 (54.8%)	2806 (51.3%)	595 (63.1%)	235 (64.9%)		3745 (51.4%)	1223 (68.7%)	
Missing	26 (0.3%)	15 (0.3%)	3 (0.3%)	2 (0.6%)		20 (0.3%)	6 (0.3%)	
Smoking status								
Never smoke	3783 (41.7%)	2198 (40.2%)	408 (43.3%)	146 (40.3%)	0.221	2978 (40.9%)	805 (45.3%)	0.004
Former smoker	2255 (24.9%)	1408 (25.7%)	210 (22.3%)	103 (28.5%)		1815 (24.9%)	440 (24.7%)	
Current smoker	2900 (32.0%)	1793 (32.8%)	321 (34.0%)	109 (30.1%)		2394 (32.9%)	506 (28.4%)	
Missing	128 (1.4%)	72 (1.3%)	4 (0.4%)	4 (1.1%)		100 (1.4%)	28 (1.6%)	
Number of medications; Mean (SD)	0.746 (1.43)	0.627 (1.28)	0.603 (1.22)	0.657 (1.35)	0.992	0.707 (1.37)	0.904 (1.63)	0.003
Polipharmacy (>5 med)	306 (3.4%)	131 (2.4%)	17 (1.8%)	10 (2.8%)	0.671	220 (3.0%)	86 (4.8%)	<0.001
Risk of Depression	1608 (17.7%)	952 (17.4%)	188 (19.9%)	84 (23.2%)	0.017	1282 (17.6%)	326 (18.3%)	0.794
Missing	29 (0.3%)	19 (0.3%)	2 (0.2%)	1 (0.3%)		27 (0.4%)	2 (0.1%)	
Hypertension	4289 (47.3%)	2524 (46.1%)	436 (46.2%)	206 (56.9%)	0.001	3250 (44.6%)	1039 (58.4%)	<0.001
Diabetes Mellitus	1140 (12.6%)	652 (11.9%)	112 (11.9%)	64 (17.7%)	0.014	840 (11.5%)	300 (16.9%)	<0.001
Metabolic Syndrome	4129 (45.5%)	2488 (45.5%)	397 (42.1%)	176 (48.6%)	0.109	3292 (45.2%)	837 (47.0%)	0.096
Missing	449 (5.0%)	268 (4.9%)	56 (5.9%)	26 (7.2%)		334 (4.6%)	115 (6.5%)	
MMSE score baseline								
Mean (SD)	26.2 (3.83)	27.1 (2.90)	23.6 (4.68)	20.7 (5.29)	<0.001	27.6 (1.81)	19.5 (3.52)	
Missing	694 (7.7%)					413 (5.7%)	281 (15.8%)	
ACE-R score baseline								
Mean (SD)	75.1 (14.8)	79.2 (11.5)	61.0 (13.9)	50.2 (14.2)		79.7 (10.1)	53.4 (14.0)	<0.001
Missing	2274 (25.1%)					1677 (23.0%)	597 (33.6%)	
Cognitive impairment by MMSE								
Yes	1779 (19.6%)	555 (10.1%)	382 (40.5%)	246 (68.0%)	<0.001			
Missing		0 (0%)	0 (0%)	1 (0.3%)				
Die during study period	446 (4.9%)	224 (4.1%)	60 (6.4%)	36 (9.9%)	<0.001	280 (3.8%)	166 (9.3%)	<0.001

**Table 2.** HR and IRR for the risk of hospital admission, number of admissions to hospital and readmissions for people with cognitive decline based on ACE-R and MMSE scores.

	ACE-R			MMSE	
	Unimpaired	MCI	Suggestive of dementia	Unimpaired	Cognitive impairment
Hospital admission	*N* = 5471	*N* = 943	*N* = 362	*N* = 7287	*N* = 1779
Crude incidence measures					
Crude *N* events	1156	239	94	1567	530
Person-years	33,499	5472	2036	46,397	10,504
Rate × 1000 person-years	34.5	43.7	46.2	33.8	50.5
IPTW-adjusted survival analysis					
*N* events	1456.87	1651.04	1633.68	2021.29	2435.21
Mean (SD) survival time in years	8.00 (0.05)	7.80 (0.05)	7.56 (0.05)	7.99 (0.03)	7.62 (0.03)
Cumulative Incidence	0.27 (0.26–0.29)	0.30 (0.26–0.33)	0.30 (0.24–0.37)	0.27 (0.26–0.28)	0.33 (0.30–0.36)
HR (95% CI) univariate		1.17 (1.02–1.35)	1.19 (0.95–1.50)		1.27 (1.14–1.42)
HR (95% CI) adjusted*		1.13 (0.98–1.30)	1.14 (0.91–1.43)		1.10 (0.97–1.26)
Readmission	*N* = 1156	*N* = 239	*N* = 94	*N* = 1567	*N* = 530
Crude incidence measures					
Crude *N* events	128	26	14	175	78
Person-years	37,542	6245	2290	52,038	12,273
Rate × 1000 person-years	3.41	4.16	6.11	3.36	6.36
IPTW-adjusted survival analysis					
*N* events	163.12	179.42	204.41	232.67	352.11
Mean (SD) survival time in years	8.76 (0.05)	8.78 (0.05)	8.69 (0.05)	8.75 (0.04)	8.57 (0.04)
Cumulative Incidence	0.14 (0.12–0.17)	0.17 (0.09–0.24)	0.25 (0.06–0.39)	0.15 (0.13–0.17)	0.19 (0.14–0.24)
HR (95% CI) univariate		1.02 (0.66–1.57)	1.32 (0.74–2.35)		1.30 (0.98–1.74)
HR (95% CI) adjusted*		1.01 (0.93–1.11)	1.09 (0.98–1.22)		1.13 (0.82–1.56)
Number of hospital admissions	*N* = 5471	*N* = 943	*N* = 362	*N* = 7287	*N* = 1779
Mean (SD) for the entire cohort	0.39 (1.09)	0.45 (1.12)	0.53 (1.73)	0.40 (1.16)	0.56 (1.44)
Mean (SD) for people with admissions	1.81 (1.71)	1.83 (1.60)	2.19 (2.96)	1.80 (1.87)	2.06 (2.13)
IRR (95% CI) univariate		0.92 (0.80–1.05)	1.15 (0.98–1.34)		1.67 (1.43–1.96)
IRR (95% CI) adjusted*		0.88 (0.75–1.02)	0.83 (0.71–0.97)		1.40 (1.18–1.66)

For the first hospital admission, follow-up started on the date of the baseline cognitive assessment and continued until the date of their first hospital admission, death, or the end of available administrative follow-up (January 2024), whichever occurred first. Participants without a hospital admission were censored at death or the end of follow-up. For analyses of 30-day readmission, the sample was restricted to those with at least one admission during follow-up. Follow-up began at discharge from the index (or first) hospital admission and ended at the first readmission within 30 days, death, or the end of available administrative follow-up, whichever occurred first.

Because death precludes the occurrence of subsequent hospital admission, it was treated as a competing event. The primary analyses used IPTW-weighted cause-specific Cox proportional hazards models, in which individuals who died were censored at the time of death. As a sensitivity analysis, we fitted Fine-Gray competing-risk regression models to estimate subdistribution hazard ratios accounting for the competing risk of death. Loss to follow-up and migration were considered unlikely as hospital admission and mortality outcomes were obtained through linkage to regional and national routinely collected administrative records.

We tested the Proportional Hazards assumption in the primary analysis using the function cox.zph that correlates the corresponding set of scaled Schoenfeld residuals with time. Results from this analysis are available in Supplemental Table S3. As some co-variables did not satisfy the proportional hazards assumption, a Cox model with time-dependent covariates was fitted using the tt() function from the survival package in R based on Zhang et al. [23].

We used a negative binomial regression to assess the association between the level of cognitive impairment based on ACE-R and MMSE scores and the total number of hospital admissions. As the outcome variable was zero-inflated (as 76.4% of the cohort had no admission to hospital during the follow-up), a zero-inflated negative binomial regression was used, with the follow-up time included as offset.

Covariates included in the multivariate analysis were selected based on a previous study [24]. All analyses were performed with RStudio.

## Results

### Cohort characteristics

Of the 9462 participants enrolled in the MAUCO cohort, 9066 people completed a MMSE test at baseline and were included in the MMSE analysis. Among them, 6776 also had an ACE-R test and, therefore, were included in the ACE-R analysis. The derivation of the analytical sample is illustrated in Figure S1.

The average age for the MMSE analytical sample was 53.3 (SD 9.76) years at baseline, with 54.5% of participants being female, 19.8% had less than 6 years of education, 54.8% reported regular or bad health, and 47.3% and 12.6% had a diagnosis of hypertension or diabetes, respectively. Four hundred forty-six (4.9%) participants died during the follow-up (Table 1). The average age for the ACE-R analytical sample was 52.5 (SD 9.64) years at baseline, with 54.7% being female. Participant characteristics, including education level, income, self-reported health status, and prevalence of hypertension or diabetes did not differ significantly between the two analytical samples.

### Cognitive status

The mean MMSE and ACE-R scores at baseline were 26.2 (SD 3.83; *n* = 9066) and 75.1 (SD 14.8; *n* = 6776) points, respectively. Cognitive impairment was identified in 1779 of 9066 (19.7%) participants based on their MMSE score, while 1305 of 6776 (19.3%) had some cognitive impairment based on the ACE-R: 943 (13.9%) had mild cognitive impairment, and 362 (5.3%) had an ACE-R score below 2SD of the population mean, suggestive of dementia. Among participants with ACE-R scores below 2SD, 68.0% (146 of 362) also met criteria for cognitive impairment based on the MMSE scores; meanwhile, 555 of 5471 (10.1%) participants classified as cognitively unimpaired according to the ACE-R also had a MMSE score below the cut-point for cognitive impairment (Table 1).

In both analytical samples, people with cognitive impairment were more likely to be older, and to have lower education poorer self-reported health status and a higher prevalence of hypertension and diabetes (*p* < 0.001). People with cognitive impairment based on the ACE-R scores (*n* = 6776) were more likely to have depressive symptoms based on PHQ-2 scores (*p* = 0.017), but PHQ-2 scores were not significantly different for people with and without cognitive impairment based on the MMSE test (Table 1).

### Hospital admissions

Among the 9066 participants included in the MMSE analytical sample, 2097 (23.1%) had at least one hospital admission during follow-up. The cumulative incidence of a hospital admission was 0.28 (95% CI 0.27–0.29). The mean number of admissions to hospital among those with at least one hospital admission during that time was 1.87 (SD 1.94).

Among the 6776 participants included in the ACE-R analytical sample, 1489 (22.0%) had at least one hospital admission during follow-up. The cumulative incidence of a hospital admission was 0.28 (95% CI 0.27–0.29). The average number of admissions to hospital among those with at least one hospital admission during that time was 1.84 (SD 1.81).

**Figure 1.**
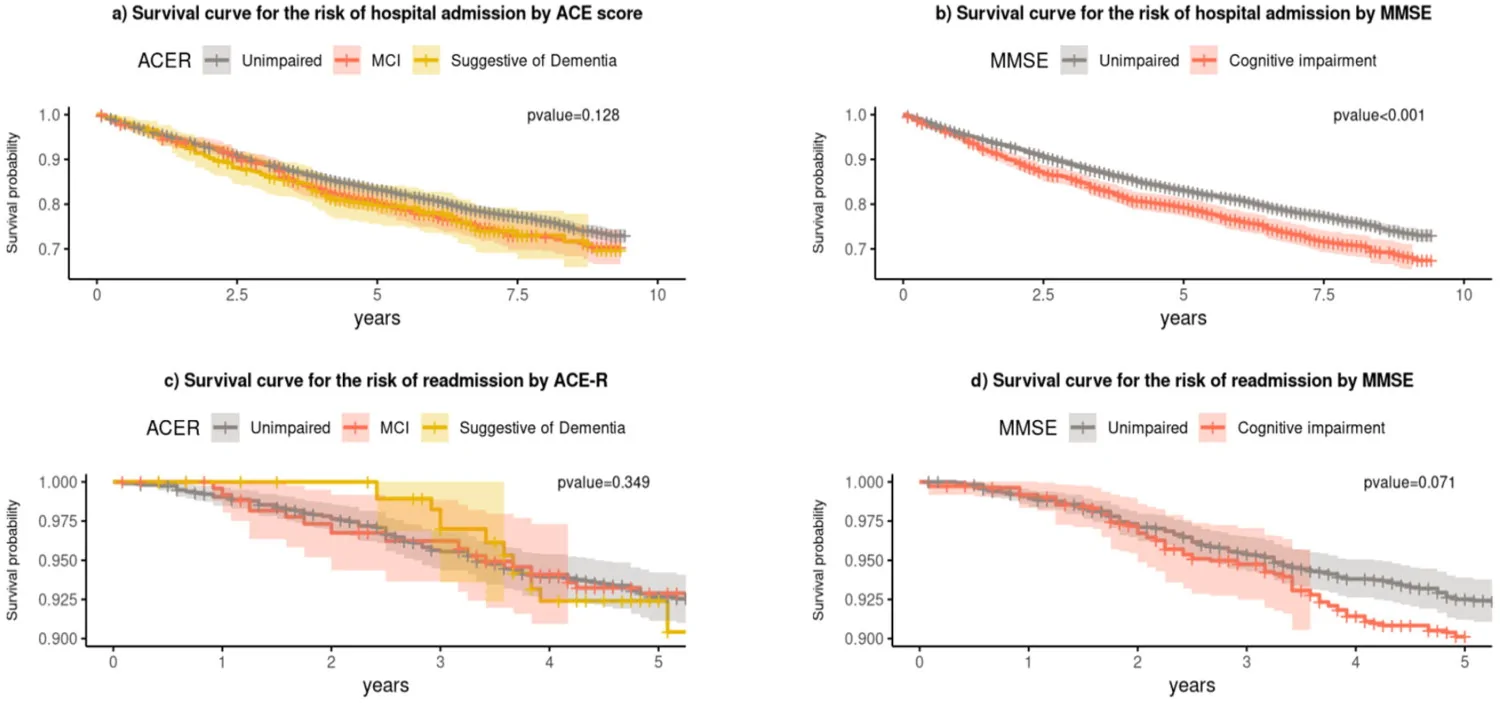
Kaplan Meir Curves for the risk of hospital admissions and readmissions by categories of ACE-R and MMSE scores.

Participants with cognitive impairment based on MMSE scores (*n* = 9066) were more likely to have had at least one hospital admission than people without cognitive impairment (Figure 1). While there was a similar trend for the ACE-R scores (*n* = 6776), the difference between groups was not significant. Participants with cognitive impairment based on MMSE scores had a 27% higher hazard of hospital admissions compared to age- and sex-weighted counterparts without cognitive impairment (HR 1.27; 95% CI 1.14–1.42). After adjusting for sociodemographic and clinical variables, the difference in the incidence of hospital admissions for people with and without cognitive impairment based on MMSE scores was not statistically significant, and having a hospital admission was largely explained by clinical variables. Participants with a diagnosis of diabetes and a low self-reported health status had 42% (HR 1.42; 95% CI 1.22–1.65) and 22% (HR 1.22; 95% CI 1.08–1.38) higher hazard of hospital admissions compared to people without diabetes or low self-reported health status (Supplementary table S4). A similar trend is observed for cognitive impairment based on ACE-R scores.

Participants with cognitive impairment based on MMSE scores had a 40% higher incidence rate of hospital admissions compared to age- and sex-weighted controls without cognitive impairment, after adjusting for sociodemographic and clinical covariates (IRR 1.40; 95% CI 1.18–1.66) (Table 2). There was no statistical difference in the rate of hospital admissions between participants with mild cognitive impairment and age- and sex-weighted controls based on ACE-R scores. However, participants with an ACE-R score below 2 SD (suggestive of dementia) had a 17% lower incidence rate of hospital admissions compared to age- and sex-weighted controls after adjusting for sociodemographic and clinical covariates (IRR 0.83; 95% CI 0.71–0.97) (Table 2).

### Hospital readmissions

Among the 2097 participants with a hospital admission, 253 (12.1%) experienced a hospital readmission within the following 30 days of discharge. The cumulative incidence of a hospital readmission was 0.17 (95% CI 0.14–0.20). Participants with cognitive impairment based on MMSE scores had a higher probability of hospital readmission during follow-up than participants without cognitive impairment, but the difference was not statistically significant (Figure 1). A similar trend was observed for the ACE-R scores. After adjusting for sociodemographic and clinical variables, the incidence of hospital readmissions was better explained by clinical variables. Participants with a diagnosis of diabetes had a 12% (HR 1.12; 95% CI 1.04–1.21) higher hazard of hospital readmissions compared to participants without diabetes (Supplementary Table S4).

## Discussion

In this population-based cohort study in adults from a rural community in Chile, we found that participants with cognitive impairment based on MMSE scores had a 40% higher incidence rate of hospital admissions compared to age- and sex-weighted controls without cognitive impairment, after adjusting for sociodemographic and clinical variables. However, when comparing participants with an ACE-R score below 2 SD, suggestive of dementia, and age- and sex-weighted controls without cognitive impairment, participants with an ACE-R score below 2 SD had a 17% lower rate of hospital admissions per unit time compared to age- and sex-weighted controls after adjusting for sociodemographic and clinical variables. The risk of hospital admission and readmission was not significantly different between participants with and without cognitive impairment after adjusting for confounders. But other clinical variables, such as a diagnosis of diabetes and self-reported health status, were more strongly associated with hospital use.

We found that participants with cognitive impairment, as defined by MMSE scores, had higher incidence rates of hospital admissions. This pattern appeared to hold for those with mild cognitive impairment, whereas the opposite trend was observed among individuals with more severe cognitive impairment. Our findings are consistent with other studies. In a cohort study conducted in the UK, people with mild cognitive impairment experienced a higher number of hospital admissions than matched controls without cognitive impairment across follow-up [25]. Another cohort study in the USA found that the severity of cognitive impairment was strongly associated with a higher incidence rate of hospital admissions, except among those with severe impairment who had a lower risk when accounting for functioning and comorbidities [26].

This lower risk observed among participants with more severe cognitive impairment as defined by the ACE-R instrument might be explained by social and healthcare system factors. Physicians may be hesitant to admit people with severe cognitive impairment to hospital settings due to limited availability of appropriate services or the presence of stigma [27,28]. However, the ACE-R incorporates the MMSE within its structure; therefore, findings based on the two cognitive measures should not be considered independent. The differences in associations found in this study may reflect differences in classification thresholds, sensitivity, and domain coverage rather than fundamentally distinct cognitive constructs. Furthermore, ACE-R data were available for a smaller subset of participants than MMSE data, and this difference in analytic samples may have introduced selection bias. Consequently, differences between MMSE- and ACE-R-based findings should be interpreted cautiously.

We did not find an association between cognitive impairment and hospital readmissions when accounting for age and comorbidities. Most of the available evidence has examined cognitive function as a predictor of readmission in the context of specific clinical conditions or procedures. Cognitive impairment has been linked to higher readmission rates among people admitted to hospital with coronary artery disease [29], heart failure [30], diabetes [31], pneumonia [32], surgery [33], and stroke [34]. The extent to which cognitive function predicts hospital readmission hospital independently of other clinical factors remains unclear. A systematic review published in 2018 reported that only 4 out of 12 studies identified a positive association between cognitive impairment and hospital readmissions [35]. More recent studies conducted in Denmark, England and the USA among older adults have shown that dementia and cognitive impairment are associated with a higher risk of hospital readmission in general [36,37].

This variation in study results is likely attributable to organizational and social factors. The number and severity of comorbidities, behavioural and psychological symptoms, as well as organizational factors such as inadequate hospital discharge planning and limited interdisciplinary collaboration, are consistently reported as determinants of elevated readmission risk in this population [38,39]. The evidence suggests this effect could be more pronounced among individuals with cognitive impairment who do not have a formal diagnosis of dementia [37]. People with cognitive impairment often have a reduced capacity to adhere to treatment regimens after discharge and insufficient social or caregiver support. Unlike patients with dementia, those with cognitive impairment are less likely to receive structured caregiving support or have formal caregiving arrangements in place, which increases their vulnerability to adverse outcomes. Further research should explicitly measure and account for these organizational and social dimensions to clarify underlying mechanisms and guide the development of targeted interventions that reduce readmission risk.

We found that clinical variables such as diabetes or self-reported health status were more strongly associated with hospital admissions and readmissions than cognitive status. These findings are consistent with prior evidence showing that chronic conditions are often stronger predictors of hospital use as they are associated with acute decompensation episodes that trigger hospital admission [40]. Measures of self-rated health are strongly associated with multimorbidity [41]. Diabetes‑related complications – including hypertension, dyslipidemia, and vascular damage, may contribute both to cognitive impairment and to an increased risk of hospital admission [42]. It is possible that the clinical manifestations of these conditions (e.g. poor glycemic control, mobility limitations, or higher disease burden) are more proximate drivers of hospital use than cognitive status alone.

### Strengths and limitations

To our knowledge, this is the first study exploring hospital use among people with and without cognitive impairment in Chile and across most Latin American countries. We relied on a large population-based cohort with more than 5 years of follow-up, using clinical and validated instruments, rather than administrative claims, to assess cognitive functioning. Hospital admissions were defined using administrative records, reducing the risk of information bias.

Despite these strengths, some limitations should be acknowledged. We lacked information on the cause and type of admission (emergency or elective), which may help explain the association between cognitive impairment and hospital rates. Residual confounding cannot be ruled out. Although we adjusted for comorbidities, this does not fully account for physical decline or functioning. Additional factors, such as access to healthcare services or patients’ and families’ preferences for care, or the level of support in the community, might also influence these findings. Cognitive impairment was classified using screening instruments rather than clinically confirmed diagnoses of mild cognitive impairment or dementia. Consequently, some degree of misclassification is possible. ACE-R data were unavailable for a substantial proportion of participants, resulting in a smaller analytical sample and increasing the possibility of selection bias. Finally, although hospital admission outcomes were obtained through linkage with public hospitals serving the study population, it is possible that some admissions occurring outside the linked hospital network may not have been captured.

## Conclusions

Cognitive impairment is associated with a higher incidence rate of hospital admissions among people with mild cognitive impairment, whereas lower admission rates were observed among those with more severe impairment. Chronic conditions and self-reported health status remained the strongest predictors of hospital admissions and readmissions, suggesting that disease burden may play a more important role in hospital utilization than cognitive status alone.

## Supplementary Material

Supplemental Material

## Data Availability

The data that support the findings of this study are available from the MAUCO cohort in accordance with the local institutional review board authorization. Restrictions apply to the availability of these data, which were used under license for the current study, and so are not publicly available and cannot be shared by the authors due to ethical, privacy, and security issues. To access the data, an application needs to be submitted to the MAUCO cohort online from the MAUCO website: www.mauco.org. Additional information can be requested at contacto@mauco.org.
