# Supplementary material for: Cognitive impairment and hospital admission risk among community-dwelling adults in the MAUCO cohort
Source: Ann Med. 2026 Aug 20;58(1):2716525. doi: 10.1080/07853890.2026.2716525 (PMC13495032; doi:10.1080/07853890.2026.2716525)
Supplement: Supplementary file 1 [file IANN_A_2716525_SM2142.docx]

**Supplementary material**

**Figure S1. Flowchart for sample derivation**

**Figure S2. Covariate balance before and after weighting for age and sex in the ACE-R and MMSE models**

**Figure S3. Distributional balance between ACE-R and MMSE categories for age and sex after weighting**

**Table S1. Balance summary across ACE-R categories for all co-variables after weighting.**

|  | Type | Max.Diff.Un | Max.Diff.Adj |
| --- | --- | --- | --- |
| Age at baseline | Contin. | 0.4493 | 0.0113 |
| Age categorical | Binary | 0.1713 | 0.0248 |
| Sex | Binary | 0.0677 | 0.0023 |
| Marital status | Binary | 0.0577 | 0.0762 |
| Marital status <NA> | Binary | 0.0192 | 0.0203 |
| Years of education | Contin. | 0.1034 | 0.1785 |
| Years of education <NA> | Contin. | 0.2344 | 0.3162 |
| Monthly income | Contin. | 0.1499 | 0.1293 |
| Monthly income <NA> | Binary | 0.0067 | 0.008 |
| Self-reported health status | Binary | 0.1385 | 0.1411 |
| Self-reported health status <NA> | Binary | 0.0028 | 0.0016 |
| Smoking status | Contin. | 0.033 | 0.0311 |
| Smoking status <NA> | Binary | 0.0089 | 0.0095 |
| Number of medications | Contin. | 0.0421 | 0.0777 |
| polipharmacy | Binary | 0.0096 | 0.0087 |
| Risk of Depression | Binary | 0.0581 | 0.0812 |
| Risk of Depression <NA> | Binary | 0.0014 | 0.0015 |
| Hypertension | Binary | 0.1077 | 0.0791 |
| Diabetes Mellitus | Binary | 0.058 | 0.0394 |
| Metabolic Syndrome | Binary | 0.0762 | 0.0755 |
| Metabolic Syndrome <NA> | Binary | 0.0228 | 0.0265 |
| MMSE score baseline | Contin. | 1.4503 | 1.3633 |
| ACE-R score baseline | Contin. | 2.187 | 2.0937 |
| Cognitive impairment by MMSE | Binary | 0.58 | 0.5576 |
| Cognitive impairment by MMSE <NA> | Binary | 0.0028 | 0.005 |
| Die during study period | Binary | 0.0585 | 0.0347 |
|  |  |  |  |
| Effective sample sizes |  |  |  |
|  | Control | MCD | Dementia |
| Unadjusted | 5471 | 943 | 362 |
| Adjusted | 5449.18 | 903.23 | 313.34 |

**Table S2. Balance summary across MMSE categories for all co-variables after weighting.**

|  | Type | Diff.Un | Diff.Adj |
| --- | --- | --- | --- |
| Age at baseline | Contin. | 0.5879 | 0.0354 |
| Age categorical | Binary | 0.2061 | -0.0054 |
| Sex | Binary | -0.0229 | -0.0112 |
| Marital status | Binary | 0.0837 | 0.0758 |
| Marital status <NA> | Binary | -0.0188 | -0.0183 |
| Years of education | Contin. | -1.2287 | -1.0851 |
| Years of education <NA> | Binary | -0.0043 | -0.0042 |
| Years of education cat | Contin. | -1.1551 | -1.0239 |
| Years of education cat <NA> | Binary | -0.0043 | -0.0042 |
| Monthly income | Contin. | -0.3195 | -0.2482 |
| Monthly income <NA> | Binary | -0.0005 | 0.001 |
| Self-reported health status | Binary | 0.1744 | 0.1767 |
| Self-reported health status <NA> | Binary | 0.0006 | -0.0004 |
| Smoking status | Contin. | -0.1047 | -0.014 |
| Smoking status <NA> | Binary | 0.002 | -0.0006 |
| Number of medications | Contin. | 0.131 | 0.0096 |
| polipharmacy | Binary | 0.0182 | 0.0031 |
| Risk of Depression | Binary | 0.0069 | 0.0148 |
| Risk of Depression <NA> | Binary | -0.0026 | -0.003 |
| Hypertension | Binary | 0.138 | 0.0423 |
| Diabetes Mellitus | Binary | 0.0534 | 0.0133 |
| Metabolic Syndrome | Binary | 0.0295 | -0.0009 |
| Metabolic Syndrome <NA> | Binary | 0.0188 | 0.021 |
| MMSE score baseline | Contin. | -2.9213 | -2.8085 |
| ACE-R score baseline | Binary | 0.1013 | 0.0888 |
| Cognitive impairment by MMSE | Contin. | -2.1533 | -2.0355 |
| Cognitive impairment by MMSE <NA> | Binary | 0.1054 | 0.0911 |
| Die during study period | Binary | 0.0549 | 0.026 |
|  |  |  |  |
| Effective sample sizes |  |  |  |
|  | No | Yes |  |
| Unadjusted | 7287 | 1779 |  |
| Adjusted | 7181.79 | 1465.35 |  |

**Table S3. Multivariate Cox proportional hazard models with Propensity Score Matching and Proportional Hazard assumption test for the risk of admission and readmissions to hospital.**

|  |  | Hospital admission |  |  |  | Hospital readmission |  |  |
| --- | --- | --- | --- | --- | --- | --- | --- | --- |
|  | HR (95% CI) | chisq | df | p | HR (95% CI) | chisq | df | p |
| ACER_CAT - MCI | 1.13 (0.98-1.30) |  |  |  | 0.97 (0.62-1.51) |  |  |  |
| ACER_CAT - Dementia | 1.13 (0.90-1.42) | 2.39 | 2 | 0.302 | 1.21 (0.67-2.17) | 33.98 | 2 | 0.000 |
| Years of education | 0.97 (0.95-0.99) | 16.98 | 1 | 0.000 | 0.98 (0.92-1.04) | 5.94 | 1 | 0.015 |
| Number of medications | 1.01 (0.95-1.08) | 0.10 | 1 | 0.756 | 0.93 (0.77-1.13) | 74.57 | 1 | 0.000 |
| Risk of Depression | 0.97 (0.77-1.23) | 2.95 | 1 | 0.086 | 1.17 (0.62-2.21) | 0.48 | 1 | 0.486 |
| Hypertension | 1.54 (1.27-1.88) | 21.43 | 1 | 0.000 | 0.97 (0.59-1.59) | 34.34 | 1 | 0.000 |
| Diabetes Mellitus | 1.31 (1.01-1.71) | 0.12 | 1 | 0.733 | 1.49 (0.86-2.57) | 11.02 | 1 | 0.001 |
| Smoking status | 1.08 (0.97-1.20) | 0.48 | 1 | 0.489 | 1.20 (0.91-1.58) | 0.65 | 1 | 0.418 |
| Self-reported health status | 1.40 (1.14-1.72) | 0.18 | 1 | 0.670 | 1.48 (0.89-2.46) | 9.15 | 1 | 0.002 |
|  | GLOBAL | 44.08 | 9 | 0.000 |  | 141.16 | 9 | 0.000 |
|  |  |  |  |  |  |  |  |  |
| MMSE - Yes | 1.10 (0.97-1.26) | 3.00 | 1 | 0.083 | 1.12 (0.81-1.55) | 0.04 | 1 | 0.835 |
| Years of education | 0.98 (0.96-0.99) | 0.52 | 1 | 0.473 | 0.96 (0.92-1.00) | 1.22 | 1 | 0.269 |
| Number of medications | 1.08 (1.04-1.12) | 0.06 | 1 | 0.800 | 0.93 (0.86-1.01) | 14.65 | 1 | 0.000 |
| Risk of Depression | 1.09 (0.94-1.26) | 0.80 | 1 | 0.372 | 1.01 (0.68-1.51) | 4.74 | 1 | 0.029 |
| Hypertension | 1.34 (1.18-1.53) | 19.96 | 1 | 0.000 | 0.98 (0.69-1.39) | 14.89 | 1 | 0.000 |
| Diabetes Mellitus | 1.40 (1.20-1.64) | 2.73 | 1 | 0.098 | 1.70 (1.19-2.43) | 10.22 | 1 | 0.001 |
| Smoking status | 1.03 (0.96-1.11) | 1.04 | 1 | 0.307 | 1.16 (0.95-1.41) | 6.63 | 1 | 0.010 |
| Self-reported health status | 1.22 (1.08-1.38) | 17.18 | 1 | 0.000 | 1.12 (0.79-1.59) | 1.23 | 1 | 0.268 |
|  | GLOBAL | 55.18 | 8 | 0.000 |  | 45.06 | 8 | 0.000 |

**Table S4. HR and IRR for hospitalization, total number of hospitalizations, and readmission with propensity score matching: full model**

|  | Hospital admission | Readmission | Number of admission |  |
| --- | --- | --- | --- | --- |
|  |  |  | Count | Zero-inflated |
|  | HR (95% CI) | HR (95% CI) | HR (95% CI) | HR (95% CI) |
| ACER_CAT - MCI | 1.13 (0.98-1.30) | 1.01 (0.93-1.11) | 0.88 (0.75-1.02) | 0.73 (0.52-1.03) |
| ACER_CAT - Dementia | 1.14 (0.91-1.43) | 1.09 (0.98-1.22) | 0.83 (0.71-0.97) | 0.54 (0.35-0.83) |
| Years of education | 0.99 (0.99-1.00) | 0.98 (0.93-1.04) | 0.95 (0.94-0.96) |  |
| Number of medications | 1.02 (0.96-1.09) | 1.01 (0.98-1.05) | 1.02 (0.98-1.06) | 0.75 (0.59-0.95) |
| Risk of Depression | 0.97 (0.77-1.22) | 1.19 (0.61-2.31) | 0.95 (0.83-1.09) |  |
| Hypertension | 1.11 (1.06-1.17) | 1.03 (0.93-1.13) | 0.85 (0.71-1.00) | 0.05 (0.02-0.13) |
| Diabetes Mellitus | 1.34 (1.03-1.75) | 1.08 (0.97-1.21) | 2.62 (2.26-3.05) |  |
| Smoking status | 1.07 (0.97-1.19) | 1.25 (0.94-1.66) | 1.26 (1.17-1.34) |  |
| Self-reported health status | 1.41 (1.16-1.73) | 1.37 (0.85-2.21) | 2.07 (1.79-2.40) | 0.95 (0.68-1.33) |
|  |  |  |  |  |
|  |  |  |  |  |
| MMSE - Yes | 1.10 (0.97-1.26) | 1.13 (0.82-1.56) | 1.40 (1.18-1.66) | 1.22 (0.65-2.31) |
| Years of education | 0.98 (0.96-0.99) | 0.96 (0.92-1.00) | 0.99 (0.97-1.01) | 1.14 (1.07-1.22) |
| Number of medications | 1.08 (1.04-1.12) | 0.99 (0.98-1.01) | 1.08 (1.04-1.13) | 0.55 (0.25-1.18) |
| Risk of Depression | 1.09 (0.94-1.26) | 1.01 (0.67-1.50) | 1.06 (0.91-1.24) |  |
| Hypertension | 1.08 (1.05-1.12) | 1.02 (0.95-1.10) | 1.29 (1.02-1.62) | 0.41 (0.23-0.72) |
| Diabetes Mellitus | 1.42 (1.22-1.65) | 1.12 (1.04-1.21) | 2.46 (2.01-3.02) | 0.16 (0.00-31.51) |
| Smoking status | 1.03 (0.96-1.10) | 1.18 (0.97-1.43) | 1.15 (1.07-1.23) |  |
| Self-reported health status | 1.22 (1.08-1.38) | 1.09 (0.77-1.54) | 2.07 (1.76-2.43) | 1.40 (0.85-2.30) |

**Table S5. HR and IRR for hospitalization, total number of hospitalizations, and readmission without propensity score matching: full model**

|  | **Hospital admission** | **Readmission** | **Number of admissions** | |
| --- | --- | --- | --- | --- |
|  |  |  | **Count** | **Zero-inflated** |
|  | **HR (95% CI)** | **HR (95% CI)** | **HR (95% CI)** | **HR (95% CI)** |
| ACER_CAT - MCI | 1.14 (0.99-1.31) | 1.03 (0.67-1.58) | 0.84 (0.62-1.14) | 0.54 (0.25-1.17) |
| ACER_CAT - Dementia | 1.11 (0.89-1.37) | 1.46 (0.84-2.56) | 0.95 (0.61-1.48) | 0.47 (0.11-2.07) |
| Sex | 0.91 (0.82-1.01) | 0.79 (0.58-1.09) | 0.73 (0.58-0.93) | 0.50 (0.30-0.83) |
| Age at baseline | 1.02 (1.02-1.03) | 1.02 (1.00-1.03) | 1.01 (1.00-1.03) | 0.93 (0.90-0.96) |
| Years of education | 0.98 (0.97-1.00) | 1.00 (0.96-1.04) | 0.96 (0.93-0.98) |  |
| Number of medications | 1.04 (1.00-1.08) | 1.00 (0.98-1.02) | 1.02 (0.95-1.1) | 0.71 (0.51-0.99) |
| Risk of Depression | 1.25 (1.10-1.43) | 0.90 (0.61-1.34) | 1.43 (1.11-1.83) |  |
| Hypertension | 1.26 (1.12-1.42) | 1.00 (0.70-1.43) | 0.88 (0.67-1.16) |  |
| Diabetes Mellitus | 1.29 (1.12-1.49) | 1.71 (1.18-2.48) | 2.29 (1.74-3.03) | 0.33 (0.16-0.67) |
| Smoking status | 1.09 (1.03-1.16) | 1.10 (0.91-1.33) | 1.26 (1.12-1.42) |  |
| Self-reported health status | 1.26 (1.13-1.41) | 1.13 (0.80-1.59) | 2.41 (1.87-3.09) | 1.68 (0.99-2.85) |
|  |  |  |  |  |
| MMSE - Yes | 1.12 (1.00-1.25) | 1.25 (0.92-1.69) | 1.47 (1.13-1.90) | 0.98 (0.53-1.80) |
| Sex | 0.88 (0.81-0.97) | 0.83 (0.64-1.08) | 0.67 (0.54-0.83) | 0.49 (0.30-0.82) |
| Age at baseline | 1.03 (1.02-1.03) | 1.02 (1.00-1.03) | 1.02 (1.01-1.03) | 0.93 (0.90-0.96) |
| Years of education | 0.98 (0.97-0.99) | 0.99 (0.96-1.03) | 1.00 (0.97-1.03) | 1.08 (1.01-1.16) |
| Number of medications | 1.05 (1.03-1.08) | 0.95 (0.88-1.02) | 1.10 (1.03-1.16) | 0.74 (0.58-0.96) |
| Risk of Depression | 1.21 (1.08-1.35) | 1.00 (0.72-1.37) | 1.33 (1.07-1.65) |  |
| Hypertension | 1.17 (1.06-1.30) | 1.05 (0.78-1.40) | 1.22 (0.95-1.55) | 0.64 (0.37-1.11) |
| Diabetes Mellitus | 1.28 (1.14-1.44) | 1.75 (1.29-2.36) | 1.78 (1.35-2.34) | 0.64 (0.25-1.65) |
| Smoking status | 1.07 (1.01-1.13) | 1.12 (0.96-1.31) | 1.32 (1.19-1.46) |  |
| Self-reported health status | 1.25 (1.14-1.38) | 1.12 (0.85-1.49) | 2.17 (1.74-2.71) | 1.44 (0.90-2.30) |

**Table S6. HR for hospitalization and readmission using Fine-Gray competing-risk regression models without propensity score matching: full model**

|  | **Hospital admission** | **Readmission** |
| --- | --- | --- |
|  |  |  |
|  | **HR (95% CI)** | **HR (95% CI)** |
| ACER_CAT - MCI | 1.13 (0.98-1.30) | 1.08 (0.71-1.65) |
| ACER_CAT - Dementia | 1.10 (0.89-1.36) | 1.37 (0.79-2.36) |
| Sex | 1.02 (1.02-1.03) | 1.03 (1.02-1.05) |
| Age at baseline | 0.92 (0.82-1.02) | 0.81 (0.60-1.11) |
| Years of education | 0.98 (0.97-1.00) | 0.98 (0.94-1.02) |
| Number of medications | 1.04 (1.00-1.08) | 0.91 (0.80-1.04) |
| Risk of Depression | 1.25 (1.10-1.43) | 1.06 (0.71-1.57) |
| Hypertension | 1.26 (1.13-1.42) | 1.37 (0.96-1.95) |
| Diabetes Mellitus | 1.29 (1.12-1.49) | 2.16 (1.48-3.14) |
| Smoking status | 1.09 (1.03-1.16) | 1.19 (0.99-1.43) |
| Self-reported health status | 1.26 (1.13-1.41) | 1.35 (0.98-1.87) |
|  |  |  |
| MMSE - Yes | 1.11 (0.99-1.24) | 1.30 (0.96-1.75) |
| Sex | 1.03 (1.02-1.03) | 1.04 (1.02-1.05) |
| Age at baseline | 0.89 (0.81-0.98) | 0.80 (0.62-1.05) |
| Years of education | 0.98 (0.97-0.99) | 0.98 (0.94-1.01) |
| Number of medications | 1.05 (1.02-1.09) | 0.99 (0.91-1.07) |
| Risk of Depression | 1.20 (1.08-1.34) | 1.12 (0.81-1.55) |
| Hypertension | 1.17 (1.06-1.30) | 1.25 (0.94-1.67) |
| Diabetes Mellitus | 1.27 (1.13-1.44) | 2.04 (1.51-2.76) |
| Smoking status | 1.07 (1.01-1.12) | 1.18 (1.01-1.37) |
| Self-reported health status | 1.25 (1.14-1.38) | 1.31 (0.99-1.72) |
